# Optimizing expectations in an internet- and mobile-based intervention for depression: study protocol for a randomized factorial trial

**DOI:** 10.1186/s13063-026-09626-2

**Published:** 2026-03-17

**Authors:** Carmen Schaeuffele, Manuel Heinrich, Stephanie Haering, Christina Kampisiou, Lara Bellu, Sebastian Burchert, Marie Puetz, Jessica Wabiszczewicz, Marcel Wilhelm, Pavle Zagorscak, Winfried Rief, Christine Knaevelsrud

**Affiliations:** 1 Department of Education and Psychology, Clinical-Psychological Intervention, Freie Universität Berlin, Habelschwerdter Allee 45, Berlin, 14195 Germany; 2 Department of Psychology, Division of Clinical Psychology and Psychotherapy, Philipps University of Marburg, Gutenbergstraße 18, Marburg, 35032 Germany; 3 German Center for Mental Health (DZPG), Partner Site Berlin-Potsdam, Potsdam, Germany

**Keywords:** Expectation, Depression, Internet- and mobile-based intervention, Cognitive behavioral, App-based

## Abstract

**Background:**

Expectations are considered an important mechanism of change in psychotherapeutic interventions, including internet- and mobile-based interventions (IMI). This study aims to investigate whether two expectation-focused microinterventions can enhance the effects of an IMI for participants with elevated depression.

**Methods:**

All participants will receive an established IMI consisting of six evidence-based cognitive behavioral modules. In this 2×3 factorial design, 720 adult participants will be randomized to receive (1) either the IMI in standard or personalized framing (first randomization factor), in combination with (2) an expectation-focused telephone call mid-treatment, a supportive phone call or no phone call (second randomization factor). The primary outcome is depression at mid- and post-treatment (BDI-II). Secondary measures include expectations, adherence, state and trait anxiety and depression, disability, negative effects, and satisfaction with treatment.

**Discussion:**

This study will increase knowledge about applying micro-interventions to modify expectations and improve the effects of IMI in the treatment of depressive symptoms. Scalable IMI has the potential to improve healthcare but ways to further improve their effects and adherence need to be explored.

**Trial registration:**

The trial was prospectively registered under DRKS00032982. Registered on July 11, 2023.

## Administrative information

Note: the numbers in curly brackets in this protocol refer to SPIRIT checklist item numbers. The order of the items has been modified to group similar items (see http://www.equator-network.org/reporting-guidelines/spirit-2013-statement-defining-standard-protocol-items-for-clinical-trials/).

**Table:** 

Title {1}	Optimizing expectations in an internet- and mobile-based intervention for depression: study protocol for a randomized factorial trial
Trial registration {2a and 2b}.	The study has been registered in the German Clinical Trials Register under DRKS00032982. Registered on July 11, 2023.
Protocol version {3}	1
Funding {4}	This study is funded by the German Research Foundation (DFG, ID: 422744262-TRR 289).
Author details {5a}	CS, MH, SH, CKa, LB, SB, MP, JW, PZ, CKn: Freie Universität Berlin, Dept. of Education and Psychology, Clinical-Psychological Intervention, Habelschwerdter Allee 45, 14195 Berlin, Germany SH, MW, WR: Department of Psychology, Division of Clinical Psychology and Psychotherapy, Philipps University of Marburg, Gutenbergstraße 18, 35032 Marburg, Germany CKn: German Center for Mental Health (DZPG), partner site Berlin-Potsdam Correspondence: carmen.schaeuffele@fu-berlin.de
Name and contact information for the trial sponsor {5b}	Freie Universität Berlin, Dept. of Education and Psychology, Clinical-Psychological Intervention, Habelschwerdter Allee 45, 14195 Berlin, Germany Philipps University of Marburg, Department of Psychology, Division of Clinical Psychology and Psychotherapy,, Gutenbergstraße 18, 35032 Marburg, Germany
Role of sponsor {5c}	The sponsor and funding body have no role in study design, data collection, analysis, interpretation or in writing of publications.

## Introduction

### Background and rationale {6a}

Depression is a highly prevalent and debilitating disorder [1]. Still, it is undertreated and access to treatment is associated with significant barriers. Internet- and mobile-based interventions (IMIs) offer a low-threshold, flexible, and easy-to-access alternative to more intensive healthcare support. IMIs are self-help interventions based on psychotherapy principles. Meta-analyses on IMIs for depression have reported medium to large effects from pre- to post-treatment [2], with comparable effects to face-to-face therapy [3]. However, traditional psychotherapy and IMIs for depression only achieve response rates of 40–50% [4]. Common side effects of psychotherapy, including increased symptoms, also frequently occur in IMIs [5]. Given the scalability and dissemination potential of IMIs, avenues to enhance their positive effects and reduce their side effects should be explored.

Optimizing expectations about the outcomes of treatments through psychological interventions may improve treatment effects. A recent meta-analysis found a positive association between early outcome expectations and treatment effects in face-to-face therapy as well as IMIs [6]. However, findings on the impact of expectations on outcome in IMIs, specifically for depression, do not yet paint a clear picture: In one study, higher expectations were associated with delayed response to an IMI for depression [7], while in another study, higher expectations predicted response [8]. The negative effect of overly optimistic expectations may be interpreted in light of cognitive prediction errors: Patients with overly optimistic early expectations may experience a discrepancy between expectations and outcomes that may impede their response to treatment (for the discussion of an optimal tipping point for expectation and expectation violation, see also Rief et al. [9]). These findings underline the need to go beyond merely inducing positive expectations, but rather need to optimize and tailor them in a personalized and realistic manner, as overly optimistic expectations may be detrimental to treatment response. Two context-specific microinterventions that have the potential to optimize expectations may be particularly relevant in IMIs: personalization and human support. Personalization is often achieved through individualized guidance but may also entail other aspects, such as tailoring the treatment content, order of content, user interaction, or alerts/reminders [10]. The interconnections between expectations and levels of personalization are not yet understood [10]. Human contact can be powerful in this digital setting, in particular for enhancing adherence rates [11]. This human contact may also present a promising avenue to optimize expectations. For example, information about the potential success of a psychological intervention could be communicated in a warm and competent manner, as this can change negative expectations, whereas simply communicating the potential benefits without a warm and competent manner does not [12].

Given their highly standardized settings, IMIs offer a unique opportunity to study the temporal dynamics and effects of expectations prospectively over longer time frames. Additionally, the IMI setting allows us to disentangle the impact of expectations from other treatment factors. Alliance with the therapist is, for instance, considered a key mechanism of change in psychological interventions. In the context of face-to-face psychotherapy, expectation-optimizing interventions are inherently intertwined with the personal contact to the therapist [13]. As therapeutic contact is limited and can be systematically controlled in IMIs, studying expectations and other treatment factors is highly informative and may provide more insight into the relative contribution of different treatment factors to the outcome, in turn facilitating a tailored intervention of specific mechanisms.

### Objectives {7}

We postulate that (simulated) personalization before initiating an IMI and an expectation-optimizing personal contact at mid-treatment would modify patients’ expectations and improve treatment effects. A factorial design will enable us to disentangle the mechanisms and effects of two important modulators of outcome expectations (i.e., personalization and personal contact) and, importantly, their temporal dynamics and interaction with person- and depression-related factors.

The objectives are:

1. To determine whether framing the IMI as personalized enhances initial expectations and amplifies treatment effects compared to standard framing.
2. To determine whether expectation-optimizing personal contact amplifies treatment effects. Here, we will also disentangle whether this effect is due to the specific quality of this contact or the non-specific, “common” factors of personal contact by comparing the expectation-optimizing contact to non-specific contact and no-contact conditions.
3. To explore which variables moderate (e.g., do patients with low expectations benefit more from an expectation-optimizing personal contact?) and/or mediate (e.g., therapeutic alliance, self-efficacy) the effects of the expectation-optimizing strategies.
4. To investigate the trajectories of depression, positive and negative expectations, and other relevant treatment factors over time.

### Trial design {8}

The proof-of-concept study uses a 2 (intervention framing pre-treatment: simulated personalization vs. standard framing) × 3 (telephone contact mid-treatment: no contact vs. non-specific contact vs. expectation-optimizing contact) factorial randomized design. Participants will be randomized to one of six intervention arms in a 1:1:1:1:1:1 allocation ratio using block randomization. This study will inspect the effects of (1) experimentally manipulating personalization expectations, as well as (2) personal contact mid-treatment, on depression and participant behavior (adherence and treatment usage).

## Methods: participants, interventions, and outcomes

### Study setting {9}

The study is jointly conducted by Freie Universität Berlin, Germany (FUB) and Philipps-University Marburg, Germany (PUM) and is part of the Collaborative Research Center (CRC)/Transregio 289 “Treatment Expectation”. The CRC 289 is an interdisciplinary collaboration project examining the impact of patient expectations on the efficacy of medical and psychological treatments. Within the present study, the intervention and all assessments will be conducted through the digital research infrastructure DIRECT. DIRECT was developed by the FUB Center for Mental Health and Digital Science as a secure environment for delivering and evaluating IMIs in clinical psychology. Interested individuals can download the DIRECT app on their smartphones to take part in the intervention and data collection. Participants will be self-selected and recruited through advertisements on social media, online media outlets, and patient advocacy networks.

### Eligibility criteria {10}

Individuals will be eligible for participation if they have a depression score indicative of a current depressive disorder, i.e., a BDI-II score ≥ 14 [15]; are ≥ 18 years old; have internet access through a smartphone; live in Germany; and are fluent in the German language. Participants will be excluded if (1) they are currently undergoing psychotherapy or will start psychotherapy within the upcoming three months; (2) report current suicidal ideation (suicidality item of the BDI-II is scored ≥ 2); (3) report current psychotic symptoms (≥ 3 questions of the DIPS psychosis screening rates as “present” [16]); (4) have had a change in psychotropic antidepressant medication within the four weeks before baseline; or (5) are medicated with benzodiazepines. The four-week window reflects the typical adjustment period following initiation or modification of antidepressant treatment, during which short-term symptom fluctuations and side effects may occur. Stabilizing medication for at least four weeks reduces potential confounding effects on depressive symptoms at baseline and during the study. Benzodiazepines will be excluded due to their acute psychotropic effects on depressive symptoms which may influence intervention effects and participants’ self-report.

### Who will take informed consent? {26a}

Participants will provide electronic informed consent within the DIRECT smartphone app.

### Additional consent provisions for collection and use of participant data and biological specimens {26b}

No biological specimens will be collected. By providing consent to participation in the present study, participants will also agree that their data may be analyzed as part of the overarching evaluation of CRC 289. Parts of the data may be shared and analyzed in combination with other subprojects of the CRC. Participants are also asked to consent to data sharing of de-identified data on a repository.

## Interventions

### Explanation for the choice of comparators {6b}

This 2 (intervention framing pre-treatment: simulated personalization vs. standard framing) × 3 (telephone contact mid-treatment: no contact vs. non-specific contact vs. expectation-optimizing contact) factorial randomized controlled trial will examine two randomization factors (framing pre-treatment and personal contact mid-treatment). The comparison is conducted between the treatment arms, considering the potential interaction between the treatment factors.

The first randomization factor concerns the framing of the intervention: Patients will either receive the intervention in the standard framing or receive the same intervention with a personalized framing, simulating that the intervention is tailored to them according to their baseline data. The second randomization factor concerns a personal contact at mid-treatment: Patients will either receive a personal contact session focused on expectations, a supportive personal contact session not focused on expectations, or no personal contact. Thus, we will determine whether modulating outcome expectations by simulating personalization and personal contact at mid-treatment contributes to the treatment outcome at 3, 6, and 12 weeks after randomization.

### Intervention description {11a}

#### IMI

All participants will receive the same IMI. The IMI utilizes established online modules that have proven effective in several large RCTs [17–19]. The IMI comprises six modules that incorporate evidence-based CBT techniques, such as expressive writing, behavioral activation, and cognitive restructuring. The IMI is not guided but contains a messaging button through which participants can reach out with a message in case of questions or technical issues. The content of each module is summarized in Table 1. We recommend a specific dosage (e.g., work ten minutes per day with the daily planner or plan one positive activity per day), but participants themselves decide how to use the provided treatment material. Participants in all treatment arms will get access to the same treatment modules.

**Table 1:** Content of each module

**General structure**
The general structure of each module is similar: Participants (1) receive standardized automatic feedback on their work with the previous module, (2) are provided with multimedia educational content on specific depressive symptoms or therapeutic techniques, and (3) are given an exercise to be completed within the module. (4) In modules 3, 4, 5 and 6 participants additionally receive an exercise to be completed daily during the following seven days (e.g., plan one positive activity per day with the planner). Between module 1, 2 and 3 participiants are given a break of three days between modules, after module 3 the break expands to seven days, offering participants enough time to practice their daily exercises
**Module 1: Expressive Writing I: Focusing on the Past**
• Psychoeducation: Depressive Symptoms (e.g., sadness, insomnia, exhaustion) • Exercise: Expressive Writing (~45 min). Participants write a letter reflecting on the circumstances surrounding the onset of their symptoms. Participants are also encouraged to reflect on coping mechanisms and strategies they used so far
**Module 2: Expressive Writing II: Focusing on the Present**
• Psychoeducation: Secondary depressive symptoms (e.g., isolation, overwhelm). Setting realistic goals • Exercise 1: Expressive Writing (~30 min). Participants write a letter reflecting on their current life situation, stressors, and depressive symptoms. Participants are also encouraged to reflect on joyful activities and ressources • Exercise 2: Goals (~30 min). Participans create a list with realistic goals they wish to achieve during the program. Participants are also asked to identify three goals that are most important to them
**Module 3: Behavioral Activation I**
• Psychoeducation: Associtation between activity and mood • Exercise 1: Collection of meaningful activities (~15 min). Participans create a list with activities that are meaningful to them • Exercise 2: Daily Planner and mood tracking (~10 min). Participants are introduced to the integrated daily planner and mood tracking. Participants are encouraged to implement one meaningful activity per day for the following seven days as well as track their mood
**Module 4: Behavioral Activation II**
• Psychoeducation: Coping planning • Exercise 1: Collection of personal barriers and coping strategies (~15 min). Participans reflect on personal barriers obstructing the implementation of meaningful activities. Participants are offered coping strategies to facilitate implementation • Exercise 2: Daily Planner with coping stegies (~10 min). Participants are encouraged to continue their work with the daily planner for the following seven days. Participants are also instructed to reflect on potential barriers while planning and to use coping stragies
**Module 5: Cognitive Reconstructuring I**
• Psychoeducation: Association between cognitions and mood • Exercise 1: Multimedia bias training (~25 min). Participants receive short sequences (video, audio or text) of ambiguous situations. Then, they are presented with three different possible cognitions (either positive, negative, or neutral), of which they choose the one most relable to them. Then, the outcome of the situation is revealed. Inbetween rating situations, participants are educated on specific cognitive biases. In the end, the app provides an automated summary and feedback, revealing either a positive, negative, or neutral bias of the particiants’ cognitions • Exercise 2: Thought record (~10 min). Participants are introduced to the integrated thought record, including (a) situation, (b) thoughts, and (c) feelings. Partipants are instructed to record their thoughts and feelings for the following seven days, especially whenever they feel emotionally activated
**Module 6: Cognitive Reconstructuring II**
• Psychoeducation: Association between alternative cognitions and mood • Exercise 1: Multimedia bias training (~30 min). Participants receive short sequences (video, audio or text) of ambiguous situations. Then, they are presented with three different possible cognitions (either positive, negative or neutral), of which they choose the one most relable to them. Then, the outcome of the situation is revealed. Inbetween rating situations, participants are educated on specific cognitive biases. In the end, the app provides an automated summary and feedback, revealing either a positive, negative or neutral bias of the particiants cognitions • Exercise 2: Extended thought record (~10 min). Participants are introduced to the extended integrated thought record, including (a) situation, (b) thoughts, (c) feelings, (d) alternative thoughts and (d) alternative feelings. Partipants are instructed to use the extended thought record for the following seven days, especially whenever they feel emotionally activated
**Module 7: Expressive Writing III: Focusing on the future**
• Psychoeducation: Relapse prevention • Exercise 1: Warning signs (~10 min). Participans create a list with their personal warning signs indicating the potential onset of a depressive episode • Exercise 2: Expressive writing (~20 min). Participants write a letter reflecting on their symptoms and feelings in the beginning of the program. Participants are also encouraged to reflect on their achievements, symptom improvement as well as coping stragies they learned during the program

The IMI utilizes modules that have been investigated in several large RCTs [17–19]

#### Personalization expectation

With the first randomization factor (2 levels), we aim to modulate expectations by simulating a personalized IMI. Participants in the “personalized” arm will receive a sham version of the IMI that they believe is tailored to their needs based on their baseline data (although it is the same intervention as in the comparison group). Specifically, participants will be informed that the study team has selected the most suitable intervention components for them. The following measures will be taken to make the simulated personalization salient:

1. Before initiating the first module, participants are informed about the group to which they were randomized. Participants in the personalized group are informed about a personalized selection of the intervention modules based on their previously entered data, as well as the concept and expected benefits of personalized interventions. Participants in the standard group will receive an informative text about receiving the intervention in a previously established version. All of this information is conveyed written in the app.
2. In addition to informing them about their group allocation and the personalized modules, we introduce the metaphor of a “key-lock-principle.” The standard group will receive no further specification through means of a metaphor.
3. For the personalized group, the picture of a key will be displayed visually along with the CBT exercises in each of the six modules to make the “key-lock-principle” salient. In the standard group, we will display a generic picture of a thought bubble (see 4. below).
4. Within each module, we will tailor the headline of the psychoeducation of the respective exercise to the allocated group. While it will read “Why am I doing this?” in the standard group, the personalized group will read the headline “Why was this selected for me?”. The provided informative text about the relevance of the exercise will be the same for both groups.
5. Finally, after each module, we will assess how participants perceived the fit of the session to their needs (“session fit”). Thus, we expect to increase participants’ perception that the intervention is indeed personalized by creating the impression of asking about the efficacy of an underlying matching procedure. For the standard group, we will ask the same question. Since we did not introduce an adaption for this group, we expect that group to perceive the question about the “session fit” as a routine form of evaluation.

#### Personal contact mid-treatment

With the second randomization factor, we will investigate the effects of different variants of personal contact mid-treatment. Participants will either receive (1) no personal contact via telephone or a phone contact that is (2) supportive without focusing on expectations or (3) expectation-optimizing. The (2) non-specific telephone calls are exclusively supportive, without touching upon expectations. Thus, the supportive contact will not have a specific topic. Instead, participants are free to share any topic or concern. The telephone counselors will be trained to respond in a non-directive, empathetic manner. However, they are instructed to avoid any interventions, including interventions that may target expectations. In the (3) expectation-optimizing contact, the phone contact will focus on the expectations of the participants. The participants will be asked about their previous experiences with the intervention and about what they expect to change until the end of the intervention. The phone counselors will be trained to analyze participant’s expectations, enhance positive and realistic expectations, and modify maladaptive or unrealistic expectations by using interventions that are useful in optimizing expectations [20], including psychoeducation, informing about the effectiveness of the interventions, or increasing participants’ sense of control, in a warm and competent manner. The non-content-related aspects of the personal contact in conditions (2) and (3) are parallelized between the arms. The duration of the telephone contacts are planned to be 20 to 30 min. Counselors are master students of clinical psychology/psychotherapy who demonstrate psychotherapeutic relationship skills (e.g., empathic listening, validation). They will receive comprehensive training before treating the first client (see Section “Strategies to improve adherence to interventions {11c}”) to ensure that the intervention is delivered in a consistent manner across all participants.

### Criteria for discontinuing or modifying allocated interventions {11b}

Participants can discontinue their study participation at any time without any consequences. If a participant stops working with the IMI, they will nevertheless be invited to complete the scientific assessments. Participants will not be reallocated to another intervention arm. Moreover, the intervention will not be modified at any stage by the study team. However, it is possible that participants in one of the two personal contact groups may not complete the mid-treatment intervention (e.g., due to organizational issues). In this case, they will receive written information and questions in the app, either in a purely supportive or expectation-optimizing manner.

### Strategies to improve adherence to interventions {11c}

#### Adherence of telephone counselors

To enhance comparability and ensure adherence to the intervention protocol and the delivery of the personal contact intervention, we have implemented several measures: (1) each telephone counselor will be trained to conduct either the expectation-optimizing or non-specific, supportive telephone calls, (2) all counselors receive a talk schedule, (3) the first 2 (more if necessary) telephone calls conducted by each counselor will be carried out jointly with the supervisor. Moreover, (4) the next 2–3 conducted calls will be rated for fidelity by an external observer. In case of insufficient fidelity, telephone counselors will receive additional, targeted training as determined on a case-by-case basis by the supervisor. (5) After this training period, telephone counselors will conduct calls independently under weekly supervision. A psychotherapist with extensive expertise in IMIs will train and supervise counselors.

During the intervention phase, 10–20% of phone calls will be audio-recorded and evaluated for fidelity to the core principles by an external observer.

For the fidelity ratings, a study-specific measure was developed based on established fidelity scales [21, 22] and adapted to the specific requirements of the telephone calls in this study. Participants receiving the contact mid-treatment will also be asked, after the phone call, to indicate in one open-ended question what the contact was about. Telephone counselors will also rate how they adhered to the talk schedule.

#### Adherence of participants

Due to the self-guided IMI format, the IMI content as well as the simulated personalization, are highly standardized across participants. To enhance participants' engagement with the intervention, they will receive a push-up notification whenever a new session or assessment is available to them. Other adherence-fostering measures include standardized automatic feedback messages after completion of every module. Adherence to the assessments will additionally be supported through monetary incentives (see Section “Plans to promote participant retention and complete follow-up {18b}”). Adherence will be monitored through the DIRECT platform, which automatically records participants' engagement with the IMI (e.g., start or completion of a session, the amount of time spent in a session, and the frequency of exercise access).

### Relevant concomitant care permitted or prohibited during the trial {11d}

Participants will be considered eligible if they enter the trial with no or stable antidepressant regimen, defined as no changes in substance or dosage for at least four weeks. Participants using benzodiazepines will be excluded because of their acute effects on depressive symptoms (e.g., mood and sleep) which may influence participants’ self-report. Concurrent psychotherapy is also not permitted (see “Eligibility criteria {10}”). To avoid the between-group comparisons being confounded by starting a new or changing an ongoing treatment, we will discard the data of participants who indicate that they (i) started a psychotherapy or pharmacological treatment or (ii) changed the dosage or agent during the trial. We will model the missing observations in a manner that mimics a scenario in which this treatment uptake/change did not occur.

### Provisions for post-trial care {30}

N/a: There is no ancillary post-trial care or compensation.

### Outcomes {12}

#### Primary outcome

The primary outcome measure is depression, as assessed by the Beck Depression Inventory-II (BDI-II) [23, 24]. Change from baseline is used to describe within-group symptom change. In our main analysis which targets the difference between the treatment arms/factor levels, we consider the mean difference in BDI-II scores at the corresponding measurement occasions (3, 6, and 12 weeks after randomization) as relevant endpoints. The BDI-II is a 21-item self-report questionnaire assessing the severity of depressive symptoms over the past two weeks. Total scores range from 0 to 63. Higher scores indicate more severe depressive symptoms, with established cut-off points for minimal (0–13), mild (14–19), moderate (20–28), and severe depression (29–63). The BDI-II has demonstrated satisfactory psychometric properties, including high internal consistency and test–retest reliability, making it one of the most widely used outcome measures in psychotherapy research.

#### Secondary outcomes

All secondary outcome measures (see Table 2) have shown satisfactory reliability and validity in previous studies. In addition to the BDI-II, depression will be assessed with the 9-item Patient Health Questionnaire (PHQ-9) [25, 26]. Functioning will be assessed with the German version of the 5-item Work and Social Adjustment Scale (WSAS) [27, 28]. We will determine disability with the 7-item Pain Disability Index (PDI) [29, 30]. For sex-specific symptoms, we will assess menopausal symptoms using the Menopause Rating Scale (MRS) [31, 32]. Additionally, we will assess gender-sensitive depressive symptoms with the 25-item Gender-Sensitive Depression Screening (GSDS-25) [33]. The sex-specific symptoms will only be assessed for participants that identified with the respective sex. Personality will be assessed using the short 10-item version of the Big Five Inventory (BFI-10) [34]. We will assess satisfaction with treatment with an adapted version of the 8-item Client Satisfaction Questionnaire (CSQ-8) for IMIs [35, 36] and usability with a newly developed short scale (RAD Usability Scale). We will also ask participants to rate their goal attainment post-treatment for up to three goals they have set for themselves in module 2 of the IMI. Negative effects will be measured with the 25-item Inventory for the balanced assessment of Negative Effects of Online Interventions (INEP-ON) [37].

**Table 2:** Schedule of enrollment, interventions, and assessments

	**Study period**										
	**Enrollment**	**Baseline**	**Allocation**	**Post-allocation**							
Timepoint**	*−t*_*1*_	*t*_*0*_	**0**	*Week 1 (t*_1_**)**	*Week 2 (t*_*2*_**)**	*Week 3 (t*_*3*_*)*	*Week 4 (t*_*4*_*)*	*Week 5 (t*_*5*_*)*	*Week 6 (t*_*6*_*)*	*Week 12 (t*_*7*_*)*	*Other*
Enrollment:											
Eligibility screen	X										
Informed consent	X										
Allocation			X								
Interventions:											
Simulated Personalization				X							
Personal Contact						X					
IMI modules				X	X	X	X	X	X		
Assessments:											
Sociodemographics	X	X									
Anxiety and Depression State and Trait (STADI)		X							X		
Expectations (TEX-Q)		X									
Subjective Stress (PSS-10)		X									
Somatosensory Amplification (SSAS)		X									
Desire for Relief (self-drafted)		X									
Acceptance (UTAUT)		X									
Depression (BDI-II)	X					X			X	X	
Depression (PHQ-9)		X		X	X	X	X	X	X	X	
Functioning (WSAS)		X							X		
Disability (PDI)		X							X		
Menopausal Symptoms (MRS)		X							X	X	
Depression gender-sensitive (GSDS)		X							X		
Personality (BFI-10)		X									
Goal Attainment (GAS)									X		
Negative Effects (INEP-ON)									X		
Usability (RAD									X		
Satisfaction with Treatment (CSQ-8)									X		
Expectations (GEEE)		X		X	X	X	X	X	X		
Prior Experiences (GEEE)		X									
Treatment Effects (GEEE)									X	X	
Session Fit (self-drafted)				X	X	X	X	X	X		
Self-Efficacy (ASKU)				X	X	X	X	X	X		
Working Alliance (WAI-I)				X	X	X	X	X	X		
Subjective Adherence (self-drafted)				X	X	X	X	X	X		
Warmth & Competence of Telephone Contact (SCM)											X
Warmth & Competence of App (SCM)						X			X		

The sociodemographic variables include age, sex and gender, education, employment status, relationship status, sexual orientation, ethnicity, body measurements (height, weight, handedness), religion, disability, menstruation, and menopausal status. *ASKU*, Allgemeine Selbstwirksamkeit Kurzskala (general self-efficacy short scale); *BDI-II*, Beck Depression Inventory-II; *BFI-10*, Big Five Inventory-10; *CSQ-8*, Client Satisfaction Questionnaire-8 adapted to the IMI context; GAS, Goal Attainment; *GEEE*, Generic Assessment of Expectancies and Experiences; *GSDS*, Gender-Sensitive Depression Scale; *INEP-ON*, Inventory for the balanced assessment of Negative Effects of Psychotherapy - Online Intervention version; *MRS*, Menopause Rating Scale; *PDI*, Pain Disability Index; *PHQ-9*, Patient Health Questionnaire-9; *PSS-10*, Perceived Stress Scale-10; *SSAS*, Somatosensory Amplification Scale; *STADI*, State-Trait Anxiety-Depression Inventory; *TEX-Q*, Treatment Expectancy Questionnaire; *UTAUT*, Unified Theory of Acceptance and Use of Technology; *SCM*, Warmth and competence scales from the Stereotype Content Model; *WAI-I*, Working Alliance Inventory for Online Interventions-Short Form; *WSAS*, Work and Social Adjustment Scale; *Self-drafted*, Custom questionnaire developed for the study or CRC 289

#### Predictors

The sociodemographic variables include age, sex and gender, education, employment status, relationship status, sexual orientation, ethnicity, religion, and disability and were adapted from Ballering et al. [38] as well as Stadler et al. [39]. Additionally, we will assess body measurements (height, weight, handedness) as well as menstruation and menopausal status. Predictors include prior psychotherapy experience and the use of IMIs, assessed via the Generic Rating Scale for Previous Treatment Experiences, Outcome Expectations, and Treatment Effects (GEEE) [40]. Expectations will furthermore be assessed using the Treatment Expectation Questionnaire (TEX-Q) [41, 42]. Attitudes toward bodily sensations will be measured using the Somatosensory Amplification Scale (SSAS) [43, 44]. Anxiety and depression state and trait characteristics will be assessed using the State-Trait Anxiety-Depression Inventory (STADI) [45]. Subjective stress, including aspects of hopelessness and self-efficacy, will be measured using the 10-item Perceived Stress Scale (PSS-10) [46, 47]. To evaluate technology acceptance, we will apply the Unified Theory of Acceptance and Use of Technology (UTAUT) [48, 49]. Additionally, we will assess one item measuring the desire for relief, which was developed as part of CRC 289.

#### Psychotherapy mechanism-related constructs

We will measure expectations** (**GEEE) [40], self-efficacy [50], therapeutic alliance [51, 52], subjective adherence (translated and adapted from the item used by Smoktunowicz et al. [53]) as well as the perceived fit to this week's online module (self-drafted item) weekly. Warmth and competence of the IMI will be measured mid- and post-treatment using an adapted version of the warmth and competence scales from the Stereotype Content Model [12]. The warmth and competence of the telephone counselor will be measured following the telephone call.

#### Qualitative interviews

Twenty strategically and sequentially selected patients will also be answering questions in an in-depth semi-structured qualitative telephone interview about their experience and perceived mechanisms of change of treatment post-treatment. In selecting participants, we strive for a balance of treatment groups and gender.

### Participant timeline {13}

**Fig. 1:**
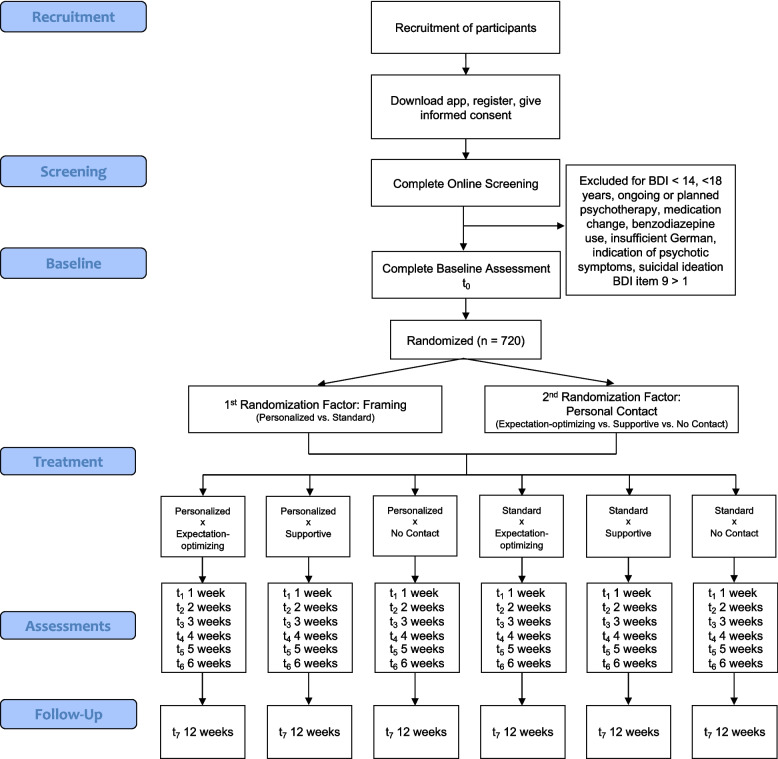
CONSORT flow diagram

Figure 1 depicts the CONSORT flowchart.

### Sample size {14}

To depict a clinically meaningful effect size of *d* = 0.35 between two arbitrary cells (e.g., personalized framing and expectation-optimizing contact vs. standard framing and no contact) in this proof-of-concept study, with an alpha of 0.05 and power of 0.80 (one-tailed, uncontrolled for multiplicity), an overall sample size of approximately 200 is needed according to G*Power [14]. The resulting sample of *N* = 600 (*n* = 100 per cell) should be sufficiently large to depict trends and inform future confirmatory trials. To consider a dropout rate of 20% per arm, we aim to recruit *N* = 720 patients (120 per treatment arm). We will include data assessed before starting the intervention as covariates to increase power.

### Recruitment {15}

Participants will be recruited through several recruitment channels. We will distribute information about the study through social media posts on an Instagram page created for this study. Selected posts will also be advertised. We will also share postings on other social media platforms as well as online discussion forums focused on mental health. It is also planned to collaborate with patient advocacy networks to increase the study's visibility. We will also cooperate with the other CRC sites to advertise the study. From all these channels, interested parties will be directed to a study website where they can learn more about the study and find instructions to download the DIRECT app.

## Assignment of interventions: allocation

### Sequence generation {16a}

An independent researcher not involved in the study team will generate a permuted block randomization list with varying block sizes using the sealed envelope web app [54].

### Concealment mechanism {16b}

The independent researcher who generated the allocation sequence will share this list with the DIRECT app developers who implement the randomization list into the intervention system. The implementation of the allocation sequence into the platform ensures that the allocation sequence is always concealed and cannot be changed by the study team.

### Implementation {16c}

Participants are self-selected, meaning they enroll themselves in the trial. Once they are considered eligible to participate (i.e., after the online eligibility screening), a member of the study team will allocate participants to one of the six groups by pushing a button in the DIRECT admin panel. The platform automatically assigns the participant to the group that is next in line based on the concealed block-randomized list stored on the platform.

## Assignment of interventions: blinding

### Who will be blinded {17a}

Due to the nature of psychotherapeutic treatment trials, full blinding is not possible. Participants will be informed that they are receiving a specific version of a digital intervention. However, they will be blind concerning the differences between the specific study groups. As study personnel delivering the mid-treatment intervention delivery (e.g., telephone call) must know the allocation to deliver the correct intervention, blinding them is not possible.

### Procedure for unblinding if needed {17b}

There is no blinding of study personnel, so unblinding is not necessary. After the “last patient in” completes the study, we will debrief participants in the simulated personalization group that they did not receive a personalized treatment.

## Data collection and management

### Plans for assessment and collection of outcomes {18a}

Our primary outcome is depressive symptoms, with secondary measures including anxiety state/trait, treatment discontinuation, adherence, usability, and negative effects. Other measures encompass sociodemographic, clinical, and psychotherapy mechanism-related variables. Outcomes will be self-reported at baseline (t_0_), mid-treatment 3 weeks after allocation (t_3_), post-treatment 6 weeks after allocation (t_6_), and at 12 weeks (t_7_) post-allocation. A shorter assessment battery of depression and psychotherapy mechanisms will be assessed weekly. All questionnaires will be collected in the DIRECT app. In case of low response rates to the questionnaires, we may contact participants via telephone to encourage completion of assessments in the app. We will also conduct qualitative interviews post-treatment with a subsample of participants to gather their experiences in the IMI and their subjective perceptions of the assumed change mechanisms. Table 2 provides a detailed overview of all assessments.

### Plans to promote participant retention and complete follow-up {18b}

The DIRECT app will automatically notify participants with push notifications that assessments are available to be completed in the app. Also, we will send reminders for mid-, post-, and follow-up assessments if participants do not complete these assessments approximately two days after availability as well as five days after availability. We will offer a monetary incentive to complete the assessments: participants will receive a €45 gift voucher for completing all assessments (mid-, post-, and follow-up). We will continue to invite participants to assessments, even if they have not continued to work with the app.

### Data management {19}

Except for the qualitative interviews, all data is gathered online and will be recorded automatically by the DIRECT app. Data will be stored on a secure server (see “Confidentiality {27}”). After completion of the study, de-identified data will be made publicly available in an Open Science Framework (OSF) repository. De-identified data will also be shared with the overarching project of the TRR/CRC 289 via a secure platform.

### Confidentiality {27}

When participants register in the DIRECT app, they provide their email address and telephone number. Participants use the email to log in, receive assessment reminders, and recover their accounts. The telephone number will enable the study team to contact participants in case of suicidal ideation/crises and for the scheduled mid-treatment telephone call. In case of low response rates to the assessments, participants may also be contacted via telephone to encourage completion of questionnaires on the platform. Data will be stored on a secure server. Different roles within the study team will ensure that only select personnel can access personal information. Personal information and questionnaire data will be stored in separate databases. The data is saved locally on the participant's smartphone. The login password as well as any other safety measures participants use on their own device ensures data. Synchronization of the local data will be achieved through encrypted (SSL) data transfer to a server located in Germany. The server is provided by Hetzner GmbH, an ISO 27001 hosting provider that complies with the EU General Data Protection Regulation (GDPR). Synchronization to the server and storage is required to ensure that the study team can support participants. The study team can access the admin panel through two-factor authentication. Data export is also protected by two-factor authentication and can only be achieved by the role of the study coordinators. Exported data is pseudonymized by assigning an internal ID (hash). Personal data is not exported. Study IDs and personal data are not matched outside of the admin panel. Following GDPR regulations, participants can delete their own data and we will also delete data upon request by participants. Upon completion of the trial, we will export all data and store it on an encrypted device for 10 years in accordance with good practice guidelines for clinical trials. After completion of the study and data analysis, all data from the app and the servers will be deleted. We will inform participants about what data is assessed, how it is transferred, and where and how long it is stored before they provide informed consent.

### Plans for collection, laboratory evaluation and storage of biological specimens for genetic or molecular analysis in this trial/future use {33}

N/a: We will not collect biological specimens.

## Statistical methods

### Statistical methods for primary and secondary outcomes {20a}

The planned analysis is described below. We will publish an updated statistical analysis plan before analyzing the research report for research question 1. This plan will take into account any issues that cannot be predicted at this stage but may emerge during the conduct of the trials (e.g., higher rates of people not receiving the intervention as planned).

Main Research Question 1: *How do simulated personalization before treatment and expectation-optimized personal contact 3 weeks after treatment initiation modulate treatment effects (e.g., the difference in depressive symptom load and secondary outcome measures at 3, 6, and 12 weeks after randomization) and treatment discontinuation?*

We assess the differences in depressive symptom load 3, 6, and 12 weeks after randomization. We treat the BDI-II score as a continuous variable. We will use factorial ANCOVA models. To explore the difference in depressive symptoms 3 weeks after treatment initiation, we use simulated personalization (F1) as the independent variable. For week 6 and 12, we use mid-treatment expectations focused contact (F2), as well as the interaction between F1 and F2 as independent variables. All ANCOVA models will include age and baseline symptom severity as covariates. We will report marginal means for each factor level of the main factors and means for each treatment arm. Pairwise comparisons between factor levels corrected for multiplicity will be reported. We will apply the same method for all other continuous secondary outcome measures available at the respective measurement occasions. We will report the difference in means in units of the scale score. Cohen’s *d*-like effect sizes will be reported. Effects on treatment discontinuation will be inspected using a rank-based test for factorial designs. Differences in rates, as well as relative effects, will be reported.

### Interim analyses {21b}

No interim analysis is planned in this trial

### Methods for additional analyses (e.g., subgroup analyses) {20b}

Beyond the question of whether and how these expectation-modulating strategies modulate treatment effects, we aim to explore the underlying mechanism. The following sub-questions are addressed:

- Sub-question 1: *How do expectations and depressive symptoms evolve?*
  We plan to compute variants of pattern-mixture models using the weekly symptom and expectation ratings as outcomes to explore whether symptoms of depression and expectations evolve differently over time. The study group is either considered by a dummy coding variable or in the form of a multi-group model. Missing data will be treated under the MAR assumption.
- Sub-question 2: Does simulated personalization enhance initial treatment expectations?
  To answer sub-question 2, a similar ANCOVA model described above, but using the baseline expectation as a covariate, will be used to explore differences in expectation at t1.
- Sub-question 3: Which variables moderate or mediate the expectation-modulating interventions?
  Regression models considering interactions between the treatment group and baseline variables will be computed. Mediator analysis will be conducted. This analysis is highly explorative. The qualitative interviews about change mechanisms will be recorded, transcribed, and analyzed using qualitative content analysis by trained staff, with an appropriate review of the assessment quality (inter-rater reliability).

### Methods in analysis to handle protocol non-adherence and any statistical methods to handle missing data {20c}

The current study is primarily interested in the effect that *could* be achieved if patients receive the expectation-modulating interventions. Therefore, we make a great effort to contact all participants by phone. Participants who do not respond to our invitation for a telephone contact will receive a session in the IBI focused on support or their expectations for the treatment. Moreover, we expect considerable variation in how participants utilize the provided IBI (i.e., they may use it at their own pace or not use the treatment modules as intended). We consider this heterogeneity as inherent to IMIs and ignore it. Data at measurement occasions of interest are mainly missing either due to (a) treatment discontinuation (i.e., participants unsubscribe from the program and, thus, can no longer be contacted by the study team and have no longer access to the treatment material) or (b) due to not completing the assessment although they still have access to the provided treatment material. For the main analysis, we plan to handle the missing measurements using multiple imputations under the missing-at-random (MAR) assumption. As a consequence, the estimate will represent the effect under the hypothetical scenario in which all randomized individuals received the assigned expectation-focused interventions and had access to the treatment material throughout the entire treatment period. We will apply the same strategy if measurements need to be discarded, such as when participants experience intercurrent events, including the initiation of psychotherapy concurrently, as this parallel treatment will confound the treatment effect of the IMI. The reasonability of the MAR assumption will be increased by including baseline as well as within treatment covariates in the imputation process. As a sensitivity analysis, a variant of a delta adjustment method will be applied (e.g., imputed values are made worse to inspect the sensitivity of the conclusions on the MAR assumption). Moreover, it will also report effects that consider that not all individuals received the expectation-optimizing manipulations. For all other analyses, missing data will be handled using either multiple imputation or full-information maximum likelihood, depending on the available data situation.

### Plans to give access to the full protocol, participant-level data and statistical code {31c}

We will make de-identified participant-level data and the statistical analysis code publicly available through a repository (OSF).

## Oversight and monitoring

### Composition of the coordinating center and trial steering committee {5d}

The trial is conducted by Freie Universität Berlin and Philipps-Universität Marburg as part of the TRR/CRC 289. While there is no independent trial steering committee, the study team meets weekly to discuss recruitment rates and any upcoming issues. In addition, a monthly meeting is held to discuss progress and milestones to ensure the trial is conducted as planned. If the study protocol warrants a significant amendment, we will obtain approval from the ethics committee of Freie Universität Berlin before implementation.

### Composition of the data monitoring committee, its role and reporting structure {21a}

An independent Data Safety and Monitoring Board (DSMB) will be established. This DSMB is independent of the study team and the funding agency. No member of the DSMB is directly involved in the study. The DSMB is composed of three researchers with expertise in the study of IMIs. The DSMB will meet annually to monitor recruitment rates, dropout rates, and adverse events.

### Adverse events and harms {22}

We will monitor suicidal ideation closely throughout the trial. If participants indicate suicidal ideation in the weekly monitoring (= 1 on the PHQ-9 item assessing suicidality) [25, 26], participants will be informed about emergency numbers and encouraged to reach out if they need additional support. If participants score > 1 on the PHQ-9 item assessing suicidality in the weekly monitoring, participants will also receive information about emergency numbers. In addition, the clinical supervisor of the trial will schedule a phone appointment to assess risks. We will ask participants in the 6- and 12-week assessments to indicate the presence of the serious adverse events life-threatening experiences, suicide attempt, and hospitalization, adapting questions used during an interview in other trials [55, 56]. Depending on the serious adverse event, appropriate measures will be taken and decided on a case-by-case basis. We will report these serious adverse events to the DSMB and summarize them in the manuscript. Safety calls and communication about adverse events will be documented online in the DIRECT client card. In the post-treatment assessment, we will also collect the presence of negative effects using the INEP-ON [37]. Besides rating the presence of negative effects, participants are further encouraged to report any additional adverse events in a free-text field. Other markers indicative of adverse events, like deterioration rates and negative effects as measured by the INEP-ON, will be reported.

### Frequency and plans for auditing trial conduct {23}

The DSMB will audit trial conduct, including recruitment rates and frequency of (serious) adverse events.

### Plans for communicating important protocol amendments to relevant parties (e.g., trial participants, ethical committees) {25}

In case of significant amendments to the study protocol, we will seek approval first from Freie Universität Berlin’s ethics committee. If approved, we will document these changes in the trial registration. We will report any amendments in the associated publications.

### Dissemination plans {31a}

We will present the findings of the trial at scientific conferences and will publish them in peer-reviewed journals. Furthermore, we will disseminate the results to the public through the science communications platforms of the TRR/CRC 289 (e.g., website, Bluesky, YouTube). We will also share de-identified data and the statistical analysis code in a data repository on OSF.

## Discussion

This RCT investigates whether expectation-focused micro-interventions can improve the effects of an IMI for adults with self-reported symptoms of depression. While in other contexts, expectation-focused interventions have been shown to improve outcomes [57], this has yet to be explored in IMIs. The goals of this study are threefold.

First, we aim to investigate the effects of tapping into expectations around personalization. Personalization is considered a way to improve treatment effects [58]. We have recently conducted a systematic review on personalization in IMIs and found generally weak evidence of the superiority of personalized over standard IMI components. Findings revealed that the implementation and communication of personalization were insufficiently detailed. This study aims to fill this gap by uncovering the underlying mechanisms behind personalization. If our study reveals that framing the IMI as personalized is sufficient to yield decreases in depressive symptoms, it could have significant clinical implications. More emphasis may be given on how to communicate treatments to patients. Future studies should then investigate whether enhanced effects in personalized treatments arise from an actual match between patients and IMI components or from expectation effects (i.e., "a treatment that is personalized to me must help me more").

Second, we aim to investigate if personal contact provided ~3 weeks after treatment initiation optimizes outcome expectations and depressive symptoms. Human support is considered important in IMIs [59]. Comparing a group with no human contact to groups that receive either expectation-focused or non-focused contact allows us to examine whether it is human contact per se or the specific expectation-optimizing strategies that optimize outcome expectations and decrease depression. Instead of more static, text-based guidance, synchronous telephone contact allows for optimizing the expectations in a personalized manner, as different levels of expectations—low, realistic, and overly optimistic expectations—and concerns may require the use of different psychotherapeutic techniques.

Third, we aim to clarify how outcome expectations prior to treatment impact outcomes but also follow their trajectory over the course of the trial and investigate their interplay with other proposed psychotherapy mechanisms. While there are some indications that expectations may not be static [60], expectations have not been investigated longitudinally within the context of IMIs yet.

### Strengths and limitations

We intend to investigate and modulate outcome expectations by two micro-interventions, using a factorial design that allows us to assess the independent and interactive effects of these micro-interventions. A key strength is the inclusion of a placebo control group for expectation-focused telephone calls, which receive warm and supportive contact without an explicit focus on expectations. This enables us to determine whether the effects are driven by the content of the telephone contact or simply by supportive human contact. Additionally, the naturalistic setting with minimal exclusion criteria ensures external validity. However, this may also result in a highly heterogenous sample with elevated depression. A limitation is that the inclusion criteria and outcome measures are all based on self-report, with no clinician rating applied. Further limitations may arise in case of low completion rates and increasing rates of individuals who do not receive the intervention as intended.

## Conclusion

This study will increase knowledge about applying micro-interventions to modify outcome expectations and improve the effects of IMI in the treatment of depressive symptoms. Scalable IMIs have the potential to improve healthcare, but ways to further improve their effects and adherence need to be explored. Our results may contribute to clarifying the role of expectation effects in the context of IMIs and identifying innovative micro-interventions that may improve their effects and adherence.

## Trial status

Participant recruitment started in July 2025 and is expected to be completed mid-2027. 

## Data Availability

The project's investigators and the investigators of the overarching projects of the CRC will have access to the final dataset. The CRC is further committed to sharing best research practices and outcomes with the other project partners (within the CRC) and the wider scientific community as defined in the Transparency and Openness Promotion Guidelines (http://cos.io/top; Level 2). We will make a de-identified dataset, as well as the analysis code, publicly available on the research data repository OSF.

## Abbreviations

ANCOVA: Analysis of covariance
ASKU: Allgemeine Selbstwirksamkeit Kurzskala (General Self-Efficacy Short Scale)
BDI-II: Beck Depression Inventory-II
BFI-10: Big Five Inventory-10
CBT: Cognitive behavioral therapy
CRC/TRR 289: Cogninitve Research Center/Transregio “Treatment Expectations”
CSQ-8: Client Satisfaction Questionnaire-8 adapted to the IMI context
DIPS: Diagnostisches Interview bei psychischen Störungen
FUB: Freie Universität Berlin
GAS: Goal Attainment Scale
GDPR: EU General Data Protection Regulation
GEEE: Generic Assessment of Expectancies and Experiences
GSDS: Gender-Sensitive Depression Scale
IMIs: Internet and mobile-based interventions
INEP-ON: Inventory for the Balanced Assessment of Negative Effects of Psychotherapy - Online Intervention version
ISO: International Organization for Standardization
MRS: Menopause Rating Scale
OSF: Open Science Framework
PDI: Pain Disability Index
PUM: Philipps-University Marburg
PHQ-9: Patient Health Questionnaire-9
PSS-10: Perceived Stress Scale-10
RCT: Randomized-Controlled Trial
SCM: Stereotype Content Model
SSAS: Somatosensory Amplification Scale
STADI: State-Trait Anxiety-Depression Inventory
TEX-Q: Treatment Expectancy Questionnaire
UTAUT: Unified Theory of Acceptance and Use of Technology
WAI-I: Working Alliance Inventory for Online Interventions-Short Form
WSAS: Work and Social Adjustment Scale
